# The International and Great Northern

**Published:** 1896-11

**Authors:** 


The Great International & Great Northern Railroad offers superior attractions to tourists to Mexico, and for the Pan-American Medical Congress which meets at the City of Mexico on the 16th inst., the management have arranged a through rate via Laredo and the Mexican National for those who wish to attend. This is the most direct, the quickest and most pleasant route from all northern, northeastern or eastern points; for roads from everywhere nearly lead to the gate of Texas—Texarkana. Entering the State at Texarkana one will strike the I. & G. N. at Longview, and the road is almost an air line—the shortest cut diagonally across the State from northeast to southwest,—Longview to Laredo, connecting there with the great Mexican National route. Advertisement and notice of which please see.

By this air line route route you make sure connections, travel in luxury, in charge of courteous officers, and you are whirled entirely across the great State of Texas—through its most beautiful sections, a splendid panorama. In buying your ticket see that it reads over the I. & G. N. R. R., and via Laredo and the Mexican National railroad. See special announcement of the I. & G. N. in our ad pages.

